# Unidirectional Evaporation‐Induced Tunable and Continuous Gradient Composite Structure for Absorption‐Dominant Electromagnetic Interference Shielding

**DOI:** 10.1002/advs.75732

**Published:** 2026-05-15

**Authors:** Dawei Zhang, Yan Zhang, Baosong Li, Abdallah Kamal, Haider Butt, Kin Liao, Lianxi Zheng

**Affiliations:** ^1^ Department of Mechanical and Nuclear Engineering Khalifa University of Science and Technology Abu Dhabi UAE; ^2^ Advanced Research and Innovation Center (ARIC) Khalifa University of Science and Technology Abu Dhabi UAE; ^3^ Machinery Industry Shanghai Lanya Petrochemical Equipment Inspection Institute Co., Ltd Shanghai China; ^4^ Department of Aerospace Engineering Khalifa University of Science and Technology Abu Dhabi UAE; ^5^ Research & Innovation Center For Graphene and 2D Materials (RIC‐2D) Khalifa University of Science and Technology Abu Dhabi UAE

**Keywords:** absorption dominance, composite foam, continuous gradient structure, electromagnetic interference shielding, evaporation

## Abstract

Absorption‐dominant electromagnetic interference (EMI) shielding requires simultaneous control of electrical conductivity and impedance matching to suppress secondary electromagnetic pollution. Here, we report a facile, scalable unidirectional evaporation method to fabricate MXene‐decorated melamine foams with a continuous, tunable electrical conductivity gradient across the thickness, free of discrete layer boundaries. Directional solvent evaporation induces controlled redistribution of MXene nanosheets within the three‐dimensional porous framework, governed by the synergistic effects of capillary, gravity, and Marangoni forces. By systematically tuning evaporation parameters, including temperature, humidity, and MXene concentration, the gradient profile can be regulated to optimize impedance matching and electromagnetic wave attenuation. The resulting composite exhibits outstanding absorption‐dominant EMI shielding performance, delivering a stable total shielding effectiveness of 53.84 dB across the X‐band with an ultralow reflection shielding effectiveness of 0.032 dB, corresponding to a high absorptivity of 0.99276. This work provides a general pathway for constructing continuous‐gradient architectures in porous composites, offering new opportunities for designing high‐performance, low‐reflection EMI shielding materials.

## Graphical abstract

A continuous conductivity gradient is formed in a porous MXene/melamine foam via directional evaporation, thereby improving impedance matching and enhancing electromagnetic attenuation. The resulting monolithic composite delivers absorption‐dominant EMI shielding with ultralow reflection, offering a simple route toward lightweight EMI shielding materials.

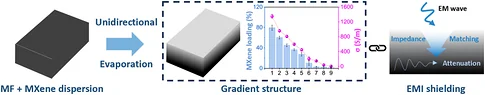

## 1 Introduction

Electromagnetic interference (EMI) has emerged as a critical challenge accompanying the rapid advancement of high‐frequency electronic devices [1, 2, 3, 4], wireless communication technologies [5, 6], and the Internet of Things [7, 8]. The increasing integration density, continuous miniaturization, and elevated operating frequencies of modern electronic systems have significantly intensified electromagnetic radiation in the surrounding environment [9, 10, 11, 12]. Uncontrolled EMI can degrade device performance by inducing signal distortion, data interference, and cross‐talk between electronic components, thereby compromising system reliability and operational stability [13, 14, 15, 16]. Moreover, growing concerns have been raised regarding the potential adverse effects of long‐term electromagnetic exposure on human health [17]. These challenges have created an urgent demand for lightweight, efficient, and reliable EMI shielding materials that can suppress electromagnetic pollution while remaining compatible with advanced electronic applications.

Traditional EMI shielding materials primarily rely on high electrical conductivity to reflect incident electromagnetic waves [18, 19, 20]. Although such reflection‐dominated mechanisms can achieve high shielding effectiveness, they often generate secondary electromagnetic pollution, which is undesirable for modern electronic systems [21]. Consequently, increasing attention has been directed toward absorption‐dominant EMI shielding materials, which dissipate electromagnetic energy within the material rather than reflecting it back into the environment [22, 23]. Achieving absorption‐dominant shielding requires a delicate balance between sufficient electrical conductivity and effective impedance matching with free space, which remains a major challenge in material design [24, 25, 26]. Porous conductive composite foams have shown great promise because of their low density, high specific surface area, and interconnected conductive networks [27, 28, 29], particularly those incorporating MXene due to the high electrical conductivity [30], large specific surface area [31], and abundant surface functional groups (─O, ─OH, and ─F) [32]. This unique porous architecture facilitates the penetration of electromagnetic waves into the material and helps reduce surface reflection. However, impedance mismatch at the air–material interface still leads to significant reflection and limits absorption efficiency [33], underscoring the importance of rational structural design.

Gradient structures have been demonstrated to be an effective strategy for addressing this challenge [34, 35, 36, 37]. By gradually varying electrical conductivity along the direction of electromagnetic wave propagation, gradient materials enable smoother impedance transitions and progressive energy dissipation. However, most reported gradient EMI shielding foams are constructed through layer‐by‐layer assembly [38, 39, 40], in which individual foam layers with different filler loadings or distinct functional components are sequentially stacked to form a laminated or stepwise gradient structure. Representative examples include asymmetric conductive‐polymer composite foams [41, 42], multilayer CNT‐based foams [43], MXene‐decorated laminated foams [35, 44], and magnetic‐electric dual‐functional layered foams [45], in which conductivity and loss mechanisms are intentionally varied across different layers to regulate impedance matching and enhance electromagnetic wave absorption. Although effective, these architectures still rely on discrete interfaces and abrupt conductivity changes, which can interrupt impedance continuity and induce additional interfacial reflection. In addition, such multistep stacking or bonding procedures may compromise structural integrity and reduce manufacturing scalability [46, 47]. Therefore, the key challenge is not only to create a gradient, but to realize a continuous, non‐stepwise gradient structure with smoothly varying conductivity across the thickness, particularly in porous composite foams. Nevertheless, such structures remain largely unexplored to date.

In this work, we address this challenge by developing a facile unidirectional evaporation method (UEM) to fabricate a monolithic composite foam with a continuous and tunable conductivity gradient across its thickness from top to bottom without distinct layer boundaries. This continuous gradient improves impedance matching at the air–material interface and promotes progressive electromagnetic attenuation within the composite, leading to absorption‐dominant EMI shielding with minimal reflection. By precisely regulating evaporation parameters, including environmental temperature, filler concentration, and humidity, the conductivity gradient within composite foams can be effectively tailored. Under the optimal condition, a stable total shielding effectiveness (SE_T_) of 53.84 dB is maintained across the X‐band, accompanied by an ultralow reflection shielding effectiveness (SE_R_) of 0.032 dB. These values correspond to a high absorptivity (A) of 0.99276 and a low reflectivity (R) of 0.00723. This study establishes a promising strategy for gradient composite foams and provides new insights into evaporation‐controlled structural design for advanced EMI shielding materials.

## 2 Experimental Section

### 2.1 Materials

Ti_3_AlC_2_ powder (MAX phase, particle size ≤40 µm) was supplied by Carbon‐Ukraine Ltd. (Ukraine). Isopropyl alcohol (IPA, C_3_H_8_O, 99%), polyvinyl alcohol (PVA), lithium fluoride (LiF, 99%), and hydrochloric acid (HCl, 37%) were purchased from Sigma‐Aldrich. Commercial melamine foams (MF) with a density of 24 kg m^−^^3^ were obtained from Shanghai Beiyou Building Materials Co., Ltd. The Polyimide (PI) film was purchased from 3 m.

### 2.2 Synthesis of Ti_3_C_2_T_x_ MXene and Dispersion Preparation

Ti_3_C_2_T_x_ MXene was synthesized using a minimally intensive layer delamination (MILD) method. Briefly, Ti_3_AlC_2_ (MAX phase) powder was first pretreated in 9 m HCl under continuous stirring for 8 h. The resulting suspension was separated by vacuum filtration and dried at 60°C for 4 h. Subsequently, an in situ HF etchant was prepared by dissolving 1.6 g of LiF in 20 mL of 9 m HCl. After complete dissolution of LiF, 1 g of the pretreated MAX powder was slowly added to the etching solution and stirred at 35°C for 24 h. The etched product was washed repeatedly with deionized (DI) water by centrifugation at 3500 rpm for 10 min per cycle until the supernatant reached a pH of approximately 6. Delaminated Ti_3_C_2_T_x_ MXene was then obtained by centrifugation at 5000 rpm for 30 min, and the stable supernatant was collected to form an aqueous MXene suspension (See Figure S1 for the characterization of the synthesized Ti_3_C_2_T_x_ MXene).

MXene dispersions with different concentrations were prepared by diluting the as‐obtained suspension with DI water. To ensure uniform dispersion, the diluted suspensions were subjected to ultrasonication (ultrasonic bath, 40 kHz, 100 W) for 30 min in an ice‐water bath to prevent overheating.

### 2.3 Preparation for MXene/MF Composite With the Gradient Structure

MF was used as the supporting substrate due to its low density and highly interconnected three‐dimensional porous architecture. The as‐received MF was sectioned into rectangular pieces with a thickness of 10 mm and lateral dimensions of 30 mm × 50 mm using a surgical blade to obtain samples with consistent geometry. The cut foams were sequentially rinsed with isopropyl alcohol and deionized (DI) water to remove residual contaminants, then oven‐dried at 60°C for 4 h.

The dried MF samples were then immersed in aqueous MXene dispersions with concentrations ranging from 1 to 11 mg mL^−^^1^. Dip‐soaking was conducted for 30 min to allow MXene nanosheets to infiltrate the porous network through capillary‐driven transport. To further facilitate MXene penetration into the internal pore walls and to eliminate trapped air, the samples were subsequently subjected to vacuum treatment for 15 min.

Following MXene deposition, the coated foam was encapsulated with a polyimide (PI) thin film on all surfaces except the bottom face, which was intentionally left exposed as the primary evaporation interface. The partially sealed sample was then placed on a supporting substrate (see Figure S2), ensuring adequate exposure of the bottom surface to ambient air while allowing controlled modulation of the evaporation conditions. When the environmental temperature was below 0°C, the entire sample rapidly froze within a short time, and the frozen sample was subsequently freeze‐dried to remove the water. After complete drying, the PI film was carefully removed, and the resulting composite was sectioned into 1 mm‐thick slices for subsequent characterization. Please see Note S1 in for the detailed description of controlling drying time, airflow, and PI sealing.

### 2.4 Characterization

The morphology and elemental distribution (Energy Dispersive Spectroscopy, EDS) of the MXene‐coated melamine foam were examined using scanning electron microscopy (SEM, JEOL JSM‐7610F). Electrical conductivity measurements were performed using a four‐point probe system (RST 8, 4 Probes Tech), and five different test locations for each sample were conducted to obtain the average value with the standard deviation. The structural characteristics of MXene were analyzed by x‐ray diffraction (XRD, Bruker D2 Phaser, Cu Kα radiation with a wavelength of 1.54184 Å), Raman spectroscopy (Horiba LabRAM HR Evolution, 633 nm excitation laser with a 100 × objective), and x‐ray photoelectron spectroscopy (XPS, Xi+ x‐ray Photoelectron Spectrometer Microprobe).

The electromagnetic (EM) parameters were evaluated using a vector network analyzer (Agilent E5071C ENA) coupled with a WR‐90 rectangular waveguide operating in the X‐band frequency range (8.2–12.4 GHz). Rectangular specimens with dimensions of 10 × 10.16 × 20.86 mm^3^ were prepared to match the waveguide aperture, ensuring reliable acquisition of the scattering parameters (S_11_, S_21_, S_22_, and S_12_). Based on the measured S‐parameters, the power coefficients of reflectivity (R), transmissivity (T), and absorptivity (A), as well as the reflection shielding effectiveness (SE_R_), absorption shielding effectiveness (SE_A_), and total electromagnetic interference (EMI) shielding effectiveness (SE_T_), were calculated according to the following relationships [14]:

$$R={\left|S11\right|}^{2}={\left|S22\right|}^{2}$$

$$T={\left|S21\right|}^{2}={\left|S12\right|}^{2}$$

A+T+R=1

SER(dB)=−10log(1−R)

SEA(dB)=−10log(T/(1−R))

$$SE_{A\;}+SE_{R}+SE_{M}=SE_{T}$$

where SE_M_ represents the contribution from multiple internal reflections, which could be neglected when SE_T_ exceeds 15 dB.

In this study, the total shielding effectiveness (SE_T_) was directly calculated from the measured S‐parameters, and the reflection (SE_R_) and absorption (SE_A_) contributions were subsequently derived from these values. SE_M_ is not independently extracted from the measurements but is considered conceptually in the theoretical framework.

## 3 Results and Discussion

### 3.1 Construction of MXene/MF Composite With Electrical Gradient Structure

**FIGURE 1:**
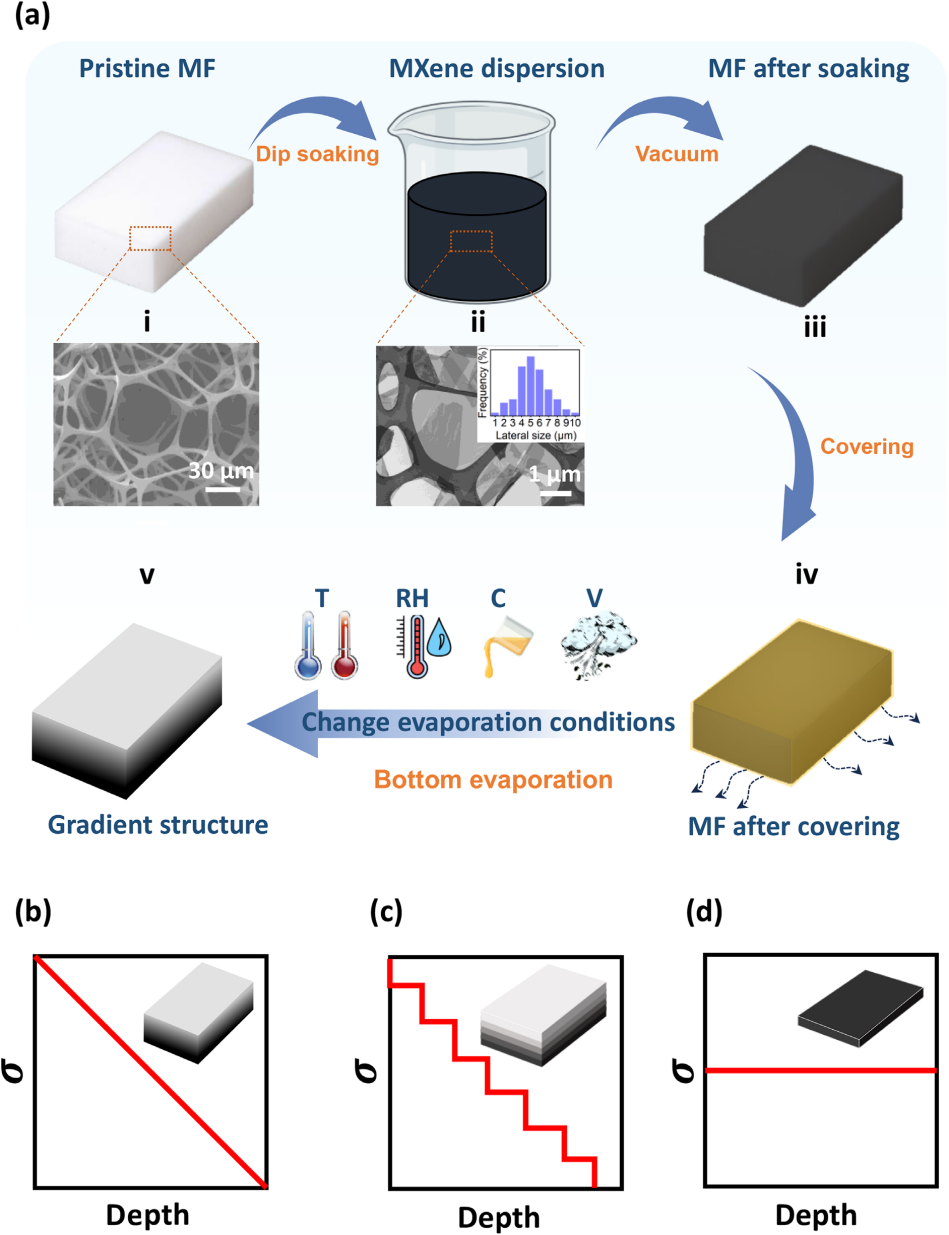
Schematic of the preparation for the evaporation‐induced gradient composite foam. (a‐i) Tailored MF bulk with 10 mm thickness and 30 mm × 50 mm lateral size. The detail view is the SEM image showing the porous microstructure of pristine MF. (a‐ii) The aqueous suspension of Ti_3_C_2_T_x_ MXene. The detail view is the TEM image of exfoliated MXene nanosheets, and the inset is the lateral size distribution of MXene nanosheets. (a‐iii) MF bulk after dip‐coating in MXene dispersion. (a‐iv) The coated MF bulk is fully covered with the Polyimide thin film except for the bottom face. (a‐v) The final product after the bottom evaporation by changing the influence factors, temperature (T), relative humidity (RH), dispersion concentration (C), and ambient wind speed (V). (b) The conductivity profile of the as‐prepared continuous gradient composite foam. (c) The stepwise conductivity profile of a control sample using the conventional layer‐by‐layer method. (d) The homogeneous conductivity of each constituent layer of the control sample.

To fabricate a continuous electrical gradient structure, pre‐cleaned MF (Figure 1a‐i) with a porous network was first immersed in a prepared MXene dispersion containing well‐delaminated nanosheets with large lateral dimensions (Figure 1a‐ii). During the dip‐soaking process, the MXene dispersion infiltrated the MF bulk and uniformly deposited onto the three‐dimensional porous framework. To ensure homogeneous impregnation and sufficient penetration throughout the foam, the MF sample, together with the MXene dispersion, was subjected to vacuum treatment for 15 min to remove trapped air within the porous structure (Figure 1a‐iii).

Following complete impregnation, the MXene/MF composite was covered with a thin polyimide (PI) film, leaving only the bottom surface exposed to ambient air (Figures 1a‐iv). Directional water evaporation was then induced from the exposed surface under controlled conditions, including temperature (T), relative humidity (RH), MXene dispersion concentration (C), and ambient airflow velocity (V). During this evaporation‐driven process, hydrogen‐bonding interactions between the MXene nanosheets and the MF skeleton promoted stable adhesion and progressively redistributed the MXene content along the thickness direction, thereby generating a continuous electrical gradient. After complete drying, the PI film was removed, yielding an MXene/MF composite (Figure 1a‐v) with a continuously graded electrical conductivity.

The resulting depth‐dependent variation in electrical conductivity (σ) is schematically illustrated in Figure 1b, where the conductivity changes smoothly and continuously along the thickness direction without distinct abruption. Such a continuous gradient enables a gradual impedance transition and uninterrupted electromagnetic energy attenuation. For comparison, a conventional gradient structure with a stepwise conductivity profile, as shown in Figure 1c, was also illustrated using a layer‐by‐layer assembly approach, in which the overall gradient is constructed by stacking multiple discrete layers with different filler loadings, and each individual layer possesses a nearly uniform electrical conductivity, as illustrated in Figure 1d. The abrupt conductivity changes at the interfaces between adjacent layers inevitably introduce discontinuities in impedance, which can lead to enhanced reflection and limit the effectiveness of electromagnetic wave absorption.

**FIGURE 2:**
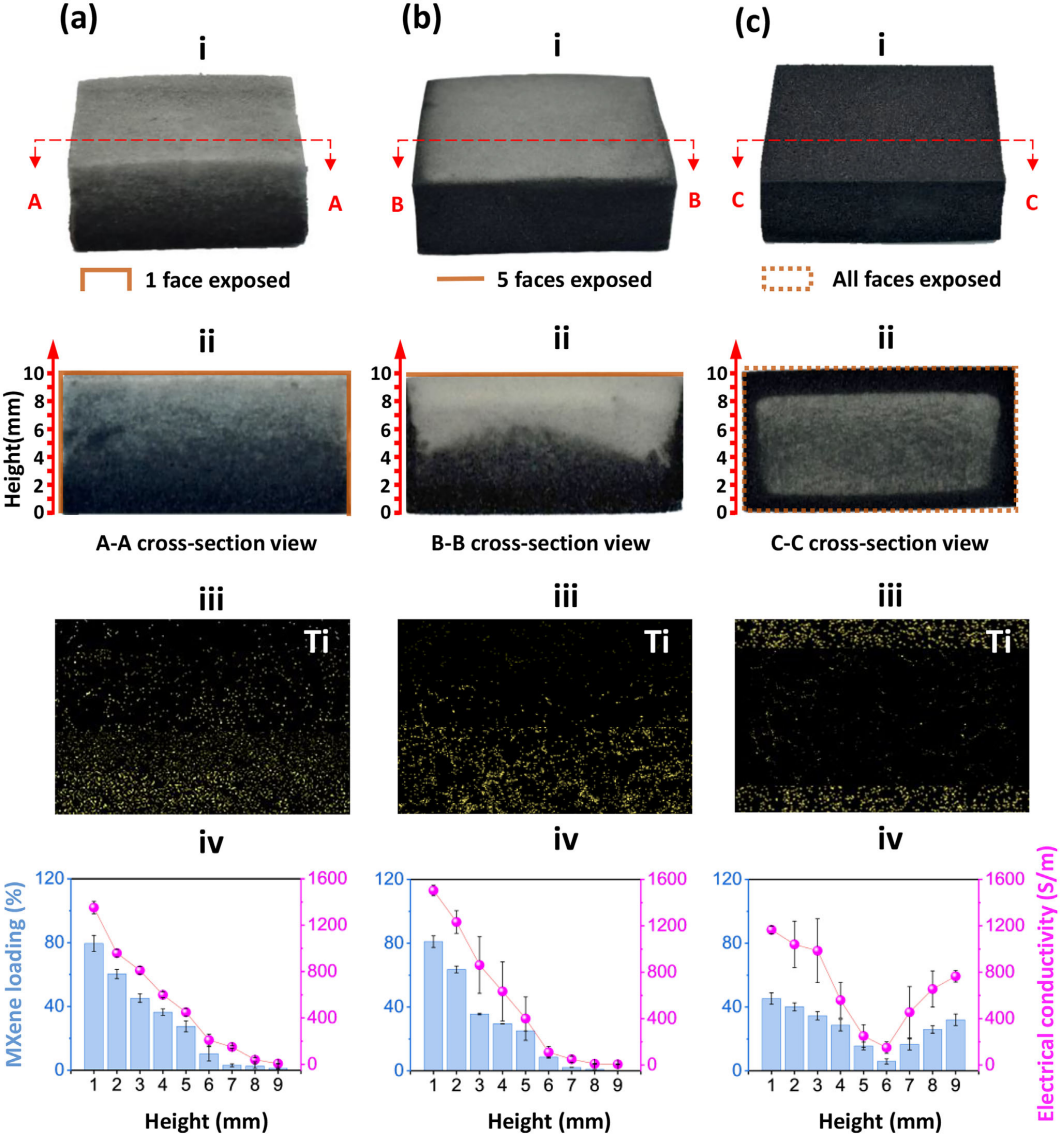
Comparison of electrical gradient profiles obtained under different exposure configurations of MF during evaporation. (a) Only the bottom face of the MF sample was exposed to ambient air, while the remaining surfaces were sealed. (b) The top face was covered, with all other faces exposed to air. (c) All faces of the sample were fully exposed, with no surface sealing. For each configuration, panel (i) shows the photograph of the as‐fabricated MXene/MF composite, (ii) presents the corresponding cross‐sectional view, (iii) displays the EDS mapping of Ti in the area of the vertical center line of the composite, and (iv) plots the electrical conductivity (pink color) and MXene loading (blue color) as a function of sample height (depth). Distinct gradient profiles are observed depending on the evaporation pathway, highlighting the critical role of directional evaporation in regulating MXene redistribution and the resulting electrical conductivity gradient.

To elucidate the influence of exposed surfaces on gradient formation in the MXene/MF composite, three distinct evaporation configurations were designed, as illustrated in Figure 2. For each configuration, the macroscopic appearance (chromatic image), elemental distribution (EDS mapping), and height or depth‐dependent electrical conductivity (σ) were systematically analyzed to evaluate the resulting gradient characteristics.

**FIGURE 3:**
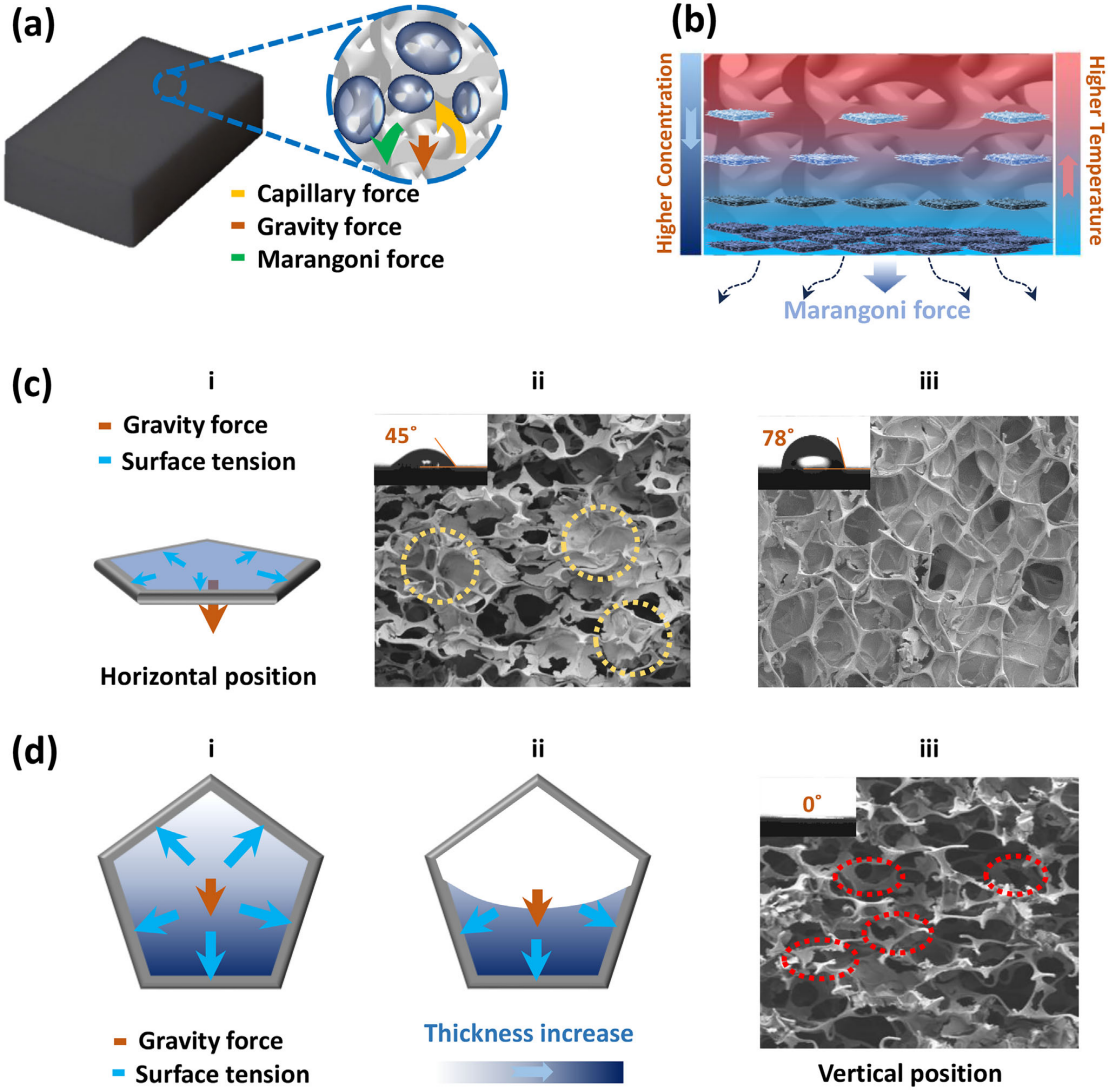
Schematic illustration of the formation mechanism of the continuous gradient structure in the composite. (a) Synergistic effects of capillary force, gravity force, and Marangoni force arising from evaporation‐induced surface tension gradients, governing the redistribution of MXene nanosheets within the porous melamine foam. (b) The mechanism of the Marangoni force formation during evaporation. (c) Formation of MXene films within horizontally oriented MF cellular structures: (c‐i) schematic illustration of MXene dispersion within horizontal MF cell skeletons during evaporation; (c‐ii) SEM image of the top surface morphology of a composite slice; and (c‐iii) SEM image of the bottom surface morphology of the bulk composite, revealing a denser MXene film, with the inset showing the corresponding water contact angle. (d) Formation of MXene films within vertically oriented MF cellular structures: (d‐i) and (d‐ii) schematic illustrations of MXene dispersion within vertical MF cell skeletons during evaporation; and (d‐iii) SEM image of the cross‐sectional surface morphology of a composite slice, with the inset showing the corresponding water contact angle, indicating enhanced surface wettability.

Figure 2a‐i presents the composite obtained using the proposed unidirectional evaporation strategy, in which only the bottom face of the MF sample was exposed to ambient air while all other surfaces were sealed. As shown in the optical image (Figure 3a‐i), the upper surface exhibits a light gray color, whereas the bottom surface appears dark black, indicating a significant difference in MXene deposition. The cross‐sectional view (Figure 2a‐ii) reveals an apparent and continuous color transition from the top to the bottom of the sample without any stepwise features, suggesting the formation of a smooth, non‐laminated gradient structure. Consistently, the EDS mapping of Ti elements (Figure 2a‐iii) illustrates a continuous gradient distribution along the thickness direction, in good agreement with the observed color variation. Moreover, the corresponding electrical conductivity (σ) profile and mass (MXene loading) increase percentage of each sectioned layer (1 mm thick) as a function of sample height (Figure 2a‐iv) provides quantitative confirmation of the gradient structure, showing a clear and continuous change in conductivity (0 to 1400 S/m) and MXene loadings along the thickness direction.

For comparison, Figure 2b‐i presents an evaporation configuration in which only the top surface of the MF sample was sealed, while the remaining faces were exposed to ambient air. Under this condition, water evaporation occurs through multiple exposed surfaces. As shown in the optical image (Figure 3b‐i), the top surface directly covered by the PI film appears grayish, whereas the other exposed faces exhibit a dark black color, indicating nonuniform MXene deposition. The corresponding cross‐sectional image (Figure 2b‐ii) reveals a pronounced interface between dark and light regions, forming a characteristic “W”‐shaped MXene distribution profile along the thickness direction. Consistently, the EDS mapping of Ti elements (Figure 2b‐iii) shows a nonuniform MXene distribution at similar depths, confirming lateral concentration variation. This heterogeneity is further reflected in the electrical conductivity profile (Figure 2b‐iv), which exhibits significant fluctuations in the interfacial region (approximately 3–5 mm), indicating uneven MXene distribution within the same depth plane.

Figure 2c‐i shows a fully exposed sample without any surface sealing, allowing unrestricted evaporation from all faces. In this case, the evaporation process becomes essentially isotropic, which suppresses the formation of a well‐defined conductivity gradient. All external surfaces appear dark black, suggesting extensive MXene accumulation near the outer regions. As evidenced by the cross‐sectional image (Figure 2c‐ii), a distinct contrast between the inner gray region and the outer dark shell with the trapezoidal shape is observed, indicating preferential MXene deposition near the exterior surfaces rather than along the thickness direction. This non‐gradient distribution is further confirmed by the EDS mapping (Figure 2c‐iii) and the corresponding conductivity profile (Figure 2c‐iv), which displays large fluctuations over a depth range of approximately 2–8 mm without a monotonic trend.

Overall, these three configurations clearly demonstrate that directional evaporation is a critical factor in regulating MXene transport and achieving a continuous electrical gradient within the porous MF matrix.

### 3.2 The Mechanism of Formation of the Gradient Structure

As illustrated in Figure 3a, the formation of the continuous gradient structure in the composite foams could arise from the synergistic action of gravity, capillary, and Marangoni forces during evaporation‐driven assembly. The proposed mechanism is described as follows.

On the one hand, owing to the foam's hydrophilic nature and its three‐dimensional, interconnected skeleton, capillary forces (yellow arrow in Figure 3a) dominate in drawing the MXene dispersion upward along the foam struts. As water is removed from the porous network, capillary pressure continuously pulls the remaining liquid upward along the internal cell walls, enabling MXene nanosheets to uniformly infiltrate and adhere to the foam skeleton. This capillary‐driven transport could promote gradual deposition of MXene along the struts rather than abrupt accumulation at discrete interfaces.

On the other hand, the gravity force (brown arrow in Figure 3a) also could act on the MXene dispersion, promoting its downward migration toward the liquid–air interface. As solvent evaporation proceeds, water molecules are continuously removed from the interface, while MXene nanosheets are retained and gradually concentrated. As water content decreases, the local MXene concentration increases, enhancing nanosheet stacking in the lower regions of the composite. In parallel, water evaporation induces both temperature and concentration gradients, resulting in surface tension gradients that generate the Marangoni force (green arrow in Figure 3a). The evaporation of water extracts heat from the interface, making the interfacial region cooler than the composite's interior. Meanwhile, the enrichment of MXene nanosheets near the evaporation front further modifies the local surface tension (Figure 3b). These temperature and concentration‐induced surface tension gradients drive interfacial liquid flow toward regions of higher surface tension, thereby contributing to the downward redistribution of the MXene dispersion.

The competition and balance among the gravity force, the capillary force, and the Marangoni force could ultimately govern the redistribution of MXene nanosheets during evaporation. While gravity and Marangoni forces favor downward migration toward the evaporation interface, capillary force counteracts this tendency by sustaining liquid transport within the porous framework. The combined effects of these three forces result in a smooth, continuous, and non‐stepwise variation in MXene deposition along the foam skeleton, leading to a continuous conductivity gradient rather than a layered structure.

During the drying process, a continuous liquid film forms along the MF skeleton due to the surface tension of the aqueous MXene dispersion, as schematically illustrated in Figure 3c‐i. In this condition, liquid films formed on horizontally oriented skeleton segments are less affected by gravity and can be maintained uniformly during evaporation. Consequently, these films remain intact throughout the drying process and form continuous, thin MXene coatings after solvent removal, as evidenced by the SEM image of the top surface morphology in Figure 3c‐ii, indicated by the yellow dashed circle. The top surface exhibits a water contact angle of approximately 45° (Figure 3c‐ii inset), which is attributed to the formation of a continuous MXene coating. Meanwhile, the bottom surface of the bulk composite shows a contact angle of approximately 78° (Figure 3c‐iii inset), indicating the formation of a dense, continuous MXene film. The observed volume shrinkage after drying (Figure S3) could also indicate the influence of the surface tension during the evaporation process.

In contrast, for vertically oriented skeleton segments, the liquid film is more influenced by gravity, leading to a gradual increase in film thickness along the gravity direction (Figure 3d‐i). As a result, a thinner liquid layer forms on the upper side of the vertical skeleton, which is prone to rupture during solvent evaporation (Figure 3d‐ii). Upon drying, this rupture produces a characteristic crescent‐shaped MXene structure, as confirmed by the SEM image of the cross‐section shown in Figure 3d‐iii, indicated by the red dashed circle. The cross‐sectional surface shows a contact angle close to 0° (Figure 3d‐iii inset), owing to its highly porous and crescent‐shaped morphology that promotes rapid water infiltration. Therefore, the final MXene distribution within the MF consists of relatively large, intact films along horizontal skeletons and smaller, crescent‐shaped deposits along vertical skeletons.

This structural differentiation between horizontal and vertical orientation plays an important role in constructing a hierarchical conductive network within the gradient composite. The continuous coatings formed along horizontal skeletons facilitate stable conductive pathways, while the discontinuous structures along vertical skeletons create microcavities at the interfaces, enhancing multiple internal scattering of electromagnetic waves, which are essential for effective electromagnetic wave attenuation.

### 3.3 The Influence of Environmental and Processing Factors on the Gradient Structure

Parameters that influence capillary, gravitational, and Marangoni forces are expected to modulate the conductivity gradient profiles of composite foams produced via unidirectional evaporation. To systematically evaluate these effects, the ambient temperature (T), relative humidity (RH), MXene dispersion concentration (C), and ambient wind speed (V) were independently varied. In each set of experiments, only one parameter was altered while the remaining variables were held constant (T = 50°C, RH = 50%, V = 0 m s^−^^1^, and C = 7 mg mL^−^^1^), enabling the isolated contribution of each factor to gradient formation to be clearly assessed.

#### 3.3.1 Effect of Temperature

**FIGURE 4:**
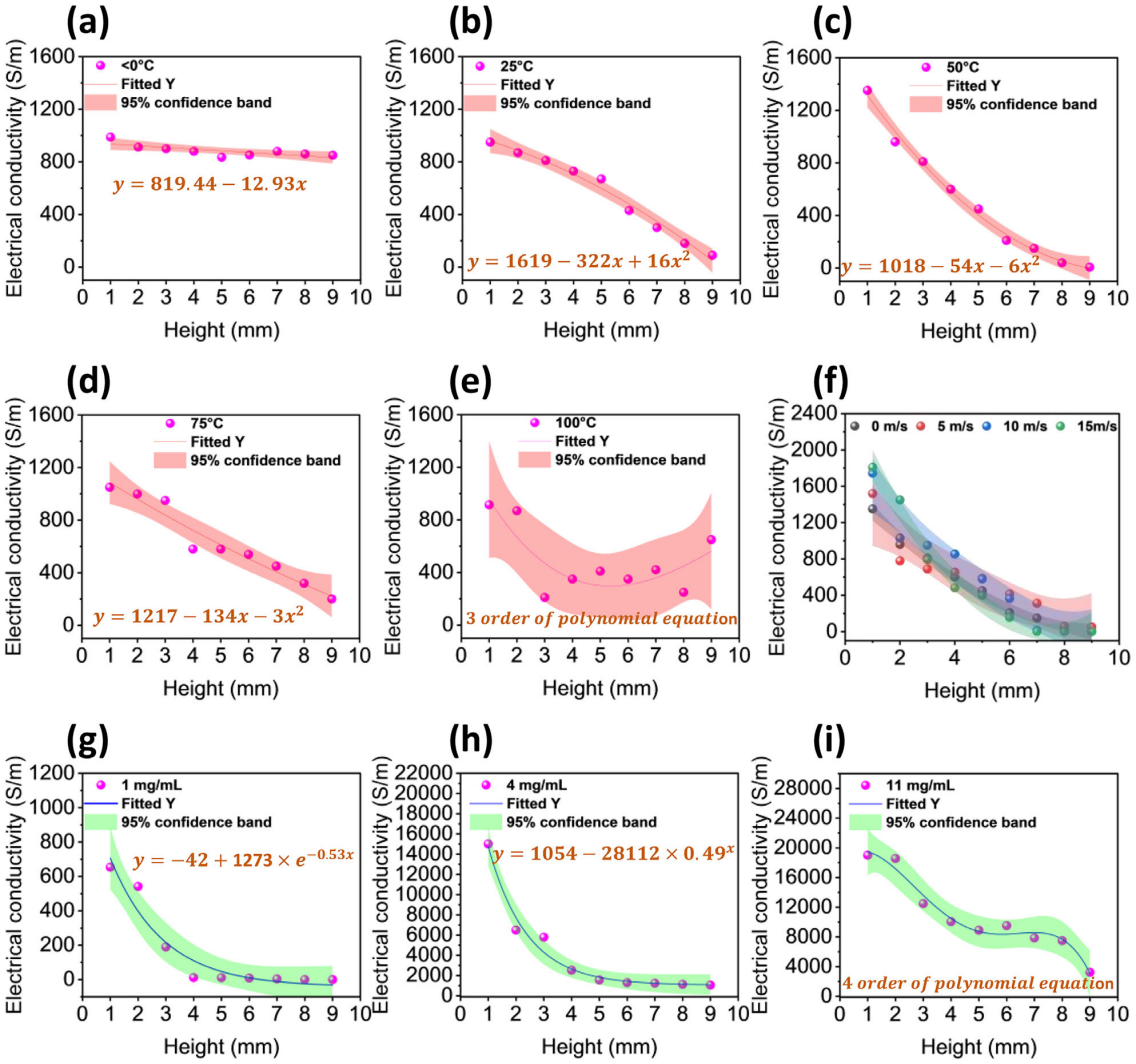
Effects of environmental and processing parameters on the conductivity gradient profiles of composite foams: (a–e) temperature T (0°C –100°C), (f) wind speed V (0–15 m s^−^^1^), and (g–i) MXene concentration C (1–11 mg mL^−^^1^). In each case, all other parameters were kept constant with T = 50°C, V = 0 m s^−^^1^, RH = 50%, and C = 7 mg mL^−^^1^. Solid lines denote fitted curves and shaded areas indicate 95% confidence intervals.

As shown in Figure 4a–e, varying the ambient temperature from 0°C to 100°C significantly modulates the electrical conductivity (σ) gradient profile of the MXene/MF composite by altering the relative contributions of the aforementioned three forces during unidirectional evaporation.

At temperatures below 0°C, the composite exhibits only minor conductivity variations along the foam thickness (Figure 4a). Under this condition, water evaporation is strongly suppressed, resulting in weak Marangoni forces and limited capillary‐driven flow. Consequently, MXene redistribution is primarily governed by slow gravity‐assisted settling, resulting in an almost linear conductivity profile with a small gradient of approximately 12.93. Despite the weak gradient, the overall conductivity remains relatively high (σ ≈ 800 S m^−^^1^) due to the minimal loss and uniform retention of MXene within the MF scaffold.

When the temperature increases to 25°C, evaporation becomes more pronounced, establishing a moderate temperature and concentration gradient near the exposed area. This induces Marangoni flow driven by surface‐tension gradients, while capillary forces actively replenish water to compensate for evaporative loss. At this temperature, the capillary force acts cooperatively with gravity and the Marangoni force, enabling controlled, gradual immobilization of MXene nanosheets. As a result, a smooth, continuous conductivity gradient develops, which can be well described by a parabolic function (y = 1018 − 54x − 6x^2^).

At 50°C, the evaporation rate is further accelerated, resulting in stronger surface‐tension gradients that intensify Marangoni transport and promote efficient MXene migration toward the exposed surface. This results in a steeper conductivity gradient than that at 25°C, with a more pronounced parabolic profile (y = 1619 − 322x + 16x^2^).

When the temperature is further increased to 75°C, the conductivity gradient becomes unstable, although an overall monotonic decreasing trend is still observed. At this elevated temperature, excessively rapid evaporation disrupts the balance between capillary flow and Marangoni‐driven transport. Strong Marangoni forces and reduced dispersion stability cause rapid local concentration buildup, while gravity‐assisted deposition accelerates MXene fixation onto the MF skeleton. This leads to nonuniform MXene accumulation and fluctuations in the conductivity profile, reflecting an imbalance among capillary, gravitational, and Marangoni forces under extreme evaporation conditions. Furthermore, when the temperature reaches 100°C, the conductivity gradient profile is severely disrupted and no longer exhibits a monotonic decrease. At this temperature, water approaches its boiling point, leading to vigorous bubble nucleation and localized boiling within the porous MF framework. The formation and collapse of vapor bubbles disrupt the continuity of the liquid phase, thereby suppressing capillary forces essential for sustained solvent replenishment. Meanwhile, the intense thermal fluctuations and rapid solvent removal destabilize surface‐tension gradients, effectively weakening Marangoni‐driven transport. Under these conditions, gravitational effects associated with bubble buoyancy and particle settling further contribute to the chaotic redistribution of MXene nanosheets (Figure S4). The combined disruption of capillary flow, Marangoni convection, and gravity‐dominated deposition results in highly irregular MXene accumulation, ultimately leading to the breakdown of a well‐defined electrical conductivity gradient.

#### 3.3.2 Effect of Wind Speed

The effect of ambient wind speed on the electrical conductivity gradient is shown in Figure 4f. Increasing the wind speed from 0 to 15 m s^−^^1^ results in a slight increase in gradient steepness; however, the overall influence remains relatively modest compared with other parameters. This indicates that wind speed plays a secondary role in regulating gradient formation. At low wind speeds, water evaporation at the exposed surface proceeds slowly, allowing sufficient time for MXene nanosheets to redistribute within the porous MF framework. As wind speed increases, it accelerates water evaporation from the exposed surface. This increase in evaporation flux strengthens Marangoni‐induced transport due to steeper concentration gradients. However, the resulting accumulation of MXene near the bottom region progressively forms a denser conductive layer, which partially shields the evaporation surface from the external airflow. This self‐limiting effect reduces the effective influence of wind speed on further evaporation enhancement. Meanwhile, capillary and gravitational effects remain stable and do not change significantly with the wind speed. Consequently, although increasing wind speed modifies the external evaporation conditions, it does not substantially alter the internal balance among capillary forces, Marangoni convection, and gravity within the porous foam. As a result, the spatial distribution of MXene and the corresponding conductivity gradient exhibit only minor variations with wind speed.

#### 3.3.3 Effect of MXene Concentration

Figure 4g–i illustrate the influence of MXene dispersion concentration on the formation of electrical conductivity gradients in the MXene/MF composite. At a low MXene concentration of 1 mg mL^−^^1^, the overall electrical conductivity remains low throughout the foam thickness, with only a weak gradient observed near the bottom region (approximately 1–3 mm). Under these conditions, capillary forces effectively draw the dilute dispersion into the porous MF scaffold; however, the limited MXene content results in an insufficient conductive network. Marangoni forces are relatively weak due to the small concentration gradient generated during evaporation, while gravitational effects dominate local MXene settling. As a result, MXene nanosheets tend to deposit in a laminated and discontinuous manner, as evidenced by the corresponding microstructural image (Figure S5), leading to a poorly developed conductivity gradient.

When the concentration is increased to 4 mg mL^−^^1^, a pronounced and continuous conductivity gradient is achieved. At this intermediate concentration, capillary infiltration efficiently transports MXene nanosheets throughout the porous framework, while evaporation‐induced concentration gradients generate sufficient Marangoni forces to drive controlled MXene redistribution toward the exposed surface. Meanwhile, gravitational effects remain secondary and do not significantly disrupt the transport process. The balanced interplay among capillary flow, Marangoni convection, and gravity enables smooth MXene immobilization along the thickness direction, resulting in a steep yet well‐distributed conductivity gradient.

At a high concentration of 11 mg mL^−^^1^, the overall conductivity increases sharply, giving rise to a highly localized conductive region. In this regime, the elevated MXene content substantially increases the dispersion viscosity, promoting rapid nanosheet stacking and network formation. These effects suppress both capillary‐driven transport and Marangoni‐induced redistribution by limiting liquid mobility (Figure S6). Concurrently, gravity‐assisted deposition becomes more prominent as MXene aggregates settle and adhere rapidly to the MF skeleton. Consequently, MXene accumulation is confined to a narrow region near the evaporation front, resulting in a gradient compression rather than a smooth conductivity profile.

#### 3.3.4 Effect of the Humidity

Relative humidity (RH) plays a critical role in regulating solvent evaporation kinetics and the formation of electrical conductivity gradients in the composite foams. By modulating the evaporation rate, RH directly influences the balance among capillary, Marangoni, and gravitational effects during unidirectional evaporation. At very low RH (5%), water evaporation proceeds extremely rapidly, which destabilizes the liquid phase within the porous MF scaffold. Under this condition, excessive evaporation induces unstable capillary flow and local rupture of the continuous liquid film, thereby interrupting both capillary‐driven transport and Marangoni‐induced redistribution. As a result, MXene migration becomes nonuniform, preventing the establishment of a well‐defined conductivity gradient, as shown in Figures S7a and S8a.

In contrast, at a high RH of 100%, solvent evaporation is strongly suppressed, and the evaporation‐induced driving force becomes insufficient to sustain effective liquid transport. The weakened evaporation rate significantly reduces surface‐tension gradients, thereby diminishing Marangoni forces, while capillary‐driven flow is also markedly reduced. Under these conditions, gravitational effects dominate MXene transport, leading to limited nanosheet migration and a relatively shallow conductivity gradient (Figures S7b and S8b). Because water evaporation was effectively halted, the sample prepared at 100% RH was characterized after freeze‐drying.

Collectively, the conductivity gradient profiles are governed by the interplay between evaporation kinetics and MXene mobility within the porous scaffold. Among all the investigated parameters, temperature and MXene concentration play the dominant roles in determining the gradient profile, while relative humidity and wind speed mainly provide auxiliary modulation. A moderate temperature (50°C) and an intermediate MXene concentration (7 mg mL^−^^1^), under 50% relative humidity and zero wind speed, are most favorable for generating smooth and continuous gradients. These findings indicate that temperature and concentration are the primary factors controlling the construction of tunable, continuous conductivity gradients.

### 3.4 EMI Shielding Performance of the Composite Foams

**FIGURE 5:**
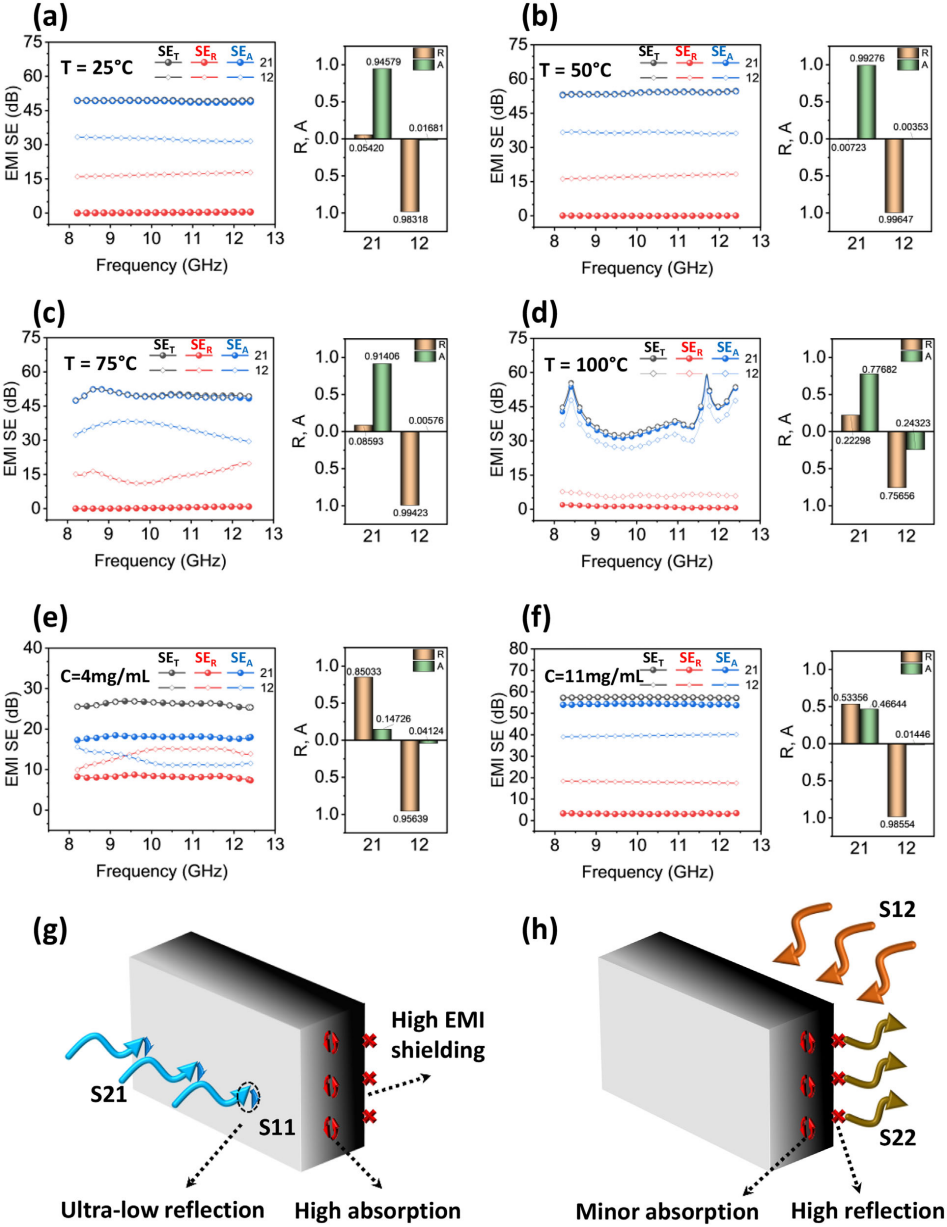
Influence of environmental and processing parameters on the EMI shielding performance (SE_A_, SE_R_, SE_T_, R, and A) of the composite foams. (a–d) Effect of ambient temperature (T = 25°C –100°C) on the EMI shielding behavior. (e–f) Effect of MXene dispersion concentration (C = 4–11 mg mL^−^^1^) on the EMI shielding performance. In all cases, the remaining parameters were kept constant (T = 50°C, V = 0 m s^−^^1^, RH = 50%, and C = 7 mg mL^−^^1^). The notation “12” indicates electromagnetic wave propagation from port 2 to port 1, while “21” represents the reverse propagation direction. (g) Schematic illustration of the EMI shielding mechanism when the electromagnetic wave is incident from port 1 to port 2. (h) Schematic illustration of the EMI shielding mechanism when the electromagnetic wave is incident from port 2 to port 1.

To evaluate the EMI shielding performance of MXene/MF composites with different electrical conductivity gradient profiles, the reflection shielding effectiveness (SE_R_), absorption shielding effectiveness (SE_A_), total EMI shielding effectiveness (SE_T_), and the corresponding power coefficients of absorptivity (A) and reflectivity (R) were measured in the X‐band frequency range. Figure 5a–f present the EMI shielding performance of composites prepared at different temperatures (25°C –100°C) and with different MXene concentrations (4–11 mg mL^−^^1^), all of which exhibit gradient structures, as shown in Figure 4.

When the incident electromagnetic wave propagates from port 1 to port 2 (Figure 5g; Figure S9) along the gradient direction from low to high conductivity, the composites exhibit low reflection and high absorption, characterized by SE_R_ < 3 dB and A > 0.5, indicating absorption‐dominant EMI shielding behavior. In contrast, when the incident direction is reversed from port 2 to port 1 (Figure 5h), the shielding performance becomes reflection‐dominant, with SE_R_ > 3 dB and A < 0.5.

With 25 and 50°C evaporation, the obtained MXene/MF composites exhibit stable EMI shielding performance across the entire X‐band, indicating that the conductivity gradient structure remains well preserved under moderate drying conditions, verified by the smooth gradient curve in Figure 4b,c. For sample evaporated at 25°C, as shown in Figure 5a, the average SE_T_ over the X band for the 21 propagation direction (following the S parameters, the notation “21” indicates electromagnetic wave propagation from port 1 to port 2, while “12” represents the reverse propagation direction) is 49.27 dB with a very low SE_R_ of 0.24 dB, whereas in the reverse 12 direction, a comparable SE_T_ of 49.31 dB is obtained but with a much higher SE_R_ of 17.74 dB. This pronounced directional difference confirms the effective regulation of impedance matching by the gradient structure. When the temperature is increased to 50°C, the average SE_T_ further improves to 53.84 dB with an extremely low SE_R_ of only 0.03 dB, corresponding to A = 0.99276 and R = 0.00723 (Figure 5b).

**FIGURE 6:**
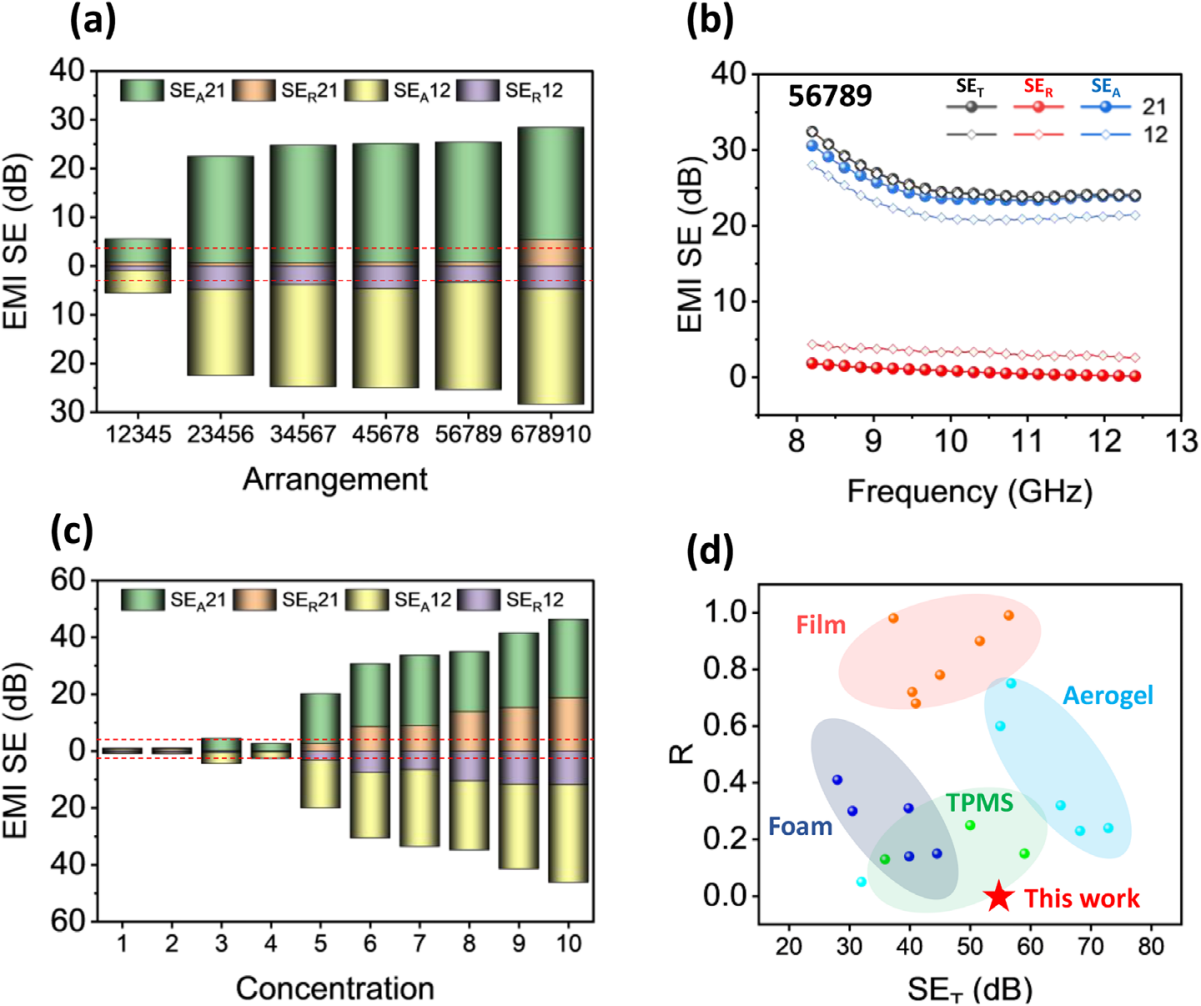
Performance comparison between the proposed unidirectional evaporation strategy, layer‐by‐layer strategy, homogeneous strategy, and previously reported MXene‐based EMI shielding architectures. (a) EMI shielding effectiveness of gradient composites fabricated using the conventional layer‐by‐layer assembly method, where the red dashed line indicates the 3 dB threshold. (b) Frequency‐dependent EMI shielding performance of the representative gradient arrangement “56789” across the X‐band. (c) EMI shielding effectiveness of homogeneous composites fabricated without a gradient structure, where the red dashed line indicates the 3 dB threshold. (d) Comparison of the absorption‐dominant shielding performance achieved in this work with previously reported films, aerogels, TPMS (Triply Periodic Minimal Surface) structures, and foams.

In contrast, when the temperature is further increased to 75°C and 100°C, the frequency‐dependent SE_T_ curves become unstable and exhibit pronounced peaks, as shown in Figures 5c,6d. At these elevated temperatures, rapid solvent evaporation significantly reduces the stability of the MXene dispersion, promoting nanosheet aggregation during drying (Figure S4b). Consequently, the resulting composites tend to form laminated or stepwise structures rather than a continuous conductivity gradient (Figure S4a). The presence of internal gaps and discrete interlayer interfaces facilitate electromagnetic wave leakage and induces cavity‐like resonance effects, which manifest as peaks in the shielding curves. As a result, although high local SE_T_ values may be observed at specific frequencies, the overall average SE_T_ decreases due to partial electromagnetic leakage.

A similar non‐monotonic relationship is observed when varying the MXene concentration. While increasing MXene loading generally enhances the total SE_T_ owing to improved electrical conductivity, electromagnetic absorption does not increase proportionally. As shown in Figure 5e, at a relatively low concentration of 4 mg mL^−^^1^, the composite exhibits an average SE_T_ of 26.2 dB and a high SER of 8.25 dB, indicating that the shielding behavior is not absorption‐dominated. This performance is attributed to the formation of a laminated structure (Figure S4), in which MXene is primarily concentrated in the bottom region of the composite, resulting in an excessively steep gradient (Figure 4g). When the MXene concentration is increased to 11 mg mL^−^^1^, the dispersion exhibits pronounced viscous flow during processing. Although a gradient structure can still form, the electrical conductivity at the top surface increases significantly to approximately 4000 S m^−^^1^ (Figure 4i). This high surface conductivity leads to severe impedance mismatch at the air–composite interface, resulting in increased reflection. Consequently, the EMI shielding SE_T_ increases to 57.25 dB with a corresponding SE_R_ of 3.31 dB, while the absorptivity decreases to A = 0.46644, indicating a transition toward reflection‐dominant shielding behavior at excessively high MXene concentrations (Figure 5f).

Overall, the optimal absorption‐dominant EMI shielding performance is achieved under a well‐balanced combination of processing temperature, MXene concentration, and gradient continuity. A moderate drying temperature of 50°C at the concentration of 7 mg mL^−^^1^ provides a favorable evaporation rate that maintains the stability of the MXene dispersion while enabling sufficient redistribution time, thereby facilitating the formation of a continuous, non‐stepwise conductivity gradient. At this temperature, excessive aggregation and premature fixation of MXene nanosheets are avoided, and impedance matching at the air–material interface is significantly improved. Meanwhile, an intermediate MXene concentration ensures adequate conductive network formation within the foam skeleton without inducing severe viscosity effects or surface overloading that would lead to reflection‐dominant behavior. Under these optimized conditions, the synergistic regulation of capillary‐driven infiltration, gravity‐assisted accumulation, and Marangoni‐induced redistribution enables smooth conductivity evolution across the composite thickness, resulting in minimal reflection, enhanced electromagnetic energy attenuation, and stable absorption‐dominant EMI shielding across the X‐band.

To evaluate the EMI shielding performance of the proposed unidirectional evaporation strategy, comparative experiments were conducted using conventional layer‐by‐layer and homogeneous coating methods. For the layer‐by‐layer approach, MF slices with a thickness of 2 mm and lateral dimensions of 30 mm × 50 mm were individually dip‐coated in as‐prepared MXene dispersions with concentrations ranging from 1 to 10 mg mL^−^^1^. After dip‐soaking with a vacuum environment, the samples were freeze‐dried, and each coated MF layer was labeled as MF1 to MF10 according to the corresponding MXene dispersion concentration.

Gradient structures were then assembled by stacking MF layers with different MXene loadings. For example, the arrangement “34567” denotes a multilayer composite composed of MF3, MF4, MF5, MF6, and MF7, in which MF3 serves as the top layer and MF7 as the bottom layer (Figure S10). The assembled composites with different layer arrangements were subsequently evaluated for EMI shielding performance. As shown in Figure 6a, the layer‐by‐layer gradient structures exhibit a maximum total shielding effectiveness of approximately 25.5 dB with absorption‐dominant behavior. Beyond this value, the shielding mechanism transitions to reflection dominance, as indicated by the red dashed line in Figure 6a. Moreover, the frequency‐dependent EMI shielding performance of the representative arrangement “56789” (Figure 6b) displays noticeable fluctuations, which are attributed to impedance mismatch and interfacial discontinuities between adjacent layers with different MXene loadings.

For comparison, homogeneous MXene‐coated MF composites were also fabricated. In this case, bulk MF with a thickness of 10 mm and lateral dimensions of 30 mm × 50 mm was dip‐coated using MXene dispersions with concentrations from 1 to 10 mg mL^−^^1^, followed by freeze‐drying. The EMI shielding performance of these homogeneous composites is summarized in Figure 6c, where the sample numbers (1–10) correspond to MXene concentrations from 1 to 10 mg mL^−^^1^. Samples prepared with MXene concentrations above 5 mg mL^−^^1^ exhibit predominantly reflection‐dominated shielding behavior, while those displaying absorption‐dominant characteristics achieve total shielding effectiveness values below 20 dB.

Compared with previously reported composites (Table S1), the composites developed in this work demonstrate substantial enhancements in both total shielding effectiveness and absorption capability (Figure 6d), while maintaining a comparably low density and a competitive specific shielding effectiveness. These results highlight the effectiveness of the proposed strategy in delivering superior EMI shielding performance while simultaneously suppressing reflection, thereby reducing secondary electromagnetic pollution.

### 3.5 EMI Shielding Mechanism of the Gradient Structure

The EMI shielding behavior of the as‐fabricated composite is governed by the synergistic effects of reflection, absorption, and multiple internal scattering [48]. When EM waves are incident on the composite, its propagation is governed by Maxwell's boundary conditions [49], which require the electric and magnetic fields at the interface to remain continuous. If the intrinsic impedances of the two media differ, the incident wave cannot simultaneously satisfy these conditions in both media, leading to partial reflection at the interface while the remaining portion is transmitted or absorbed. This impedance mismatch is therefore a fundamental origin of reflection loss in EMI shielding systems [50].

The underlying cause of impedance mismatch lies in the differences in intrinsic electromagnetic parameters, including conductivity (σ), permittivity (ε), and permeability (µ) between free space (air) and conductive materials [51]. The intrinsic impedance (*Z*) of a medium can be expressed as Equation (1) [49].

(1) $$Z=\sqrt{\frac{j\omega \mu }{\sigma +j\omega \varepsilon }}\;$$

where ω is the angular frequency, μ is the magnetic permeability, ε is the permittivity, σ is the electrical conductivity, and *j* is the imaginary unit. In free space, where σ  =  0, the intrinsic impedance reduces to $Z_{0}=\sqrt{\mu_{0}/\varepsilon_{0}}$.

In this study, the gradient structure plays a critical role in regulating impedance matching at the air–material interface by minimizing the electrical conductivity of the entry layer, allowing EM waves to effectively penetrate the interior of the material with minimal initial reflection as described in Equations (2) and (3) [52].

(2) $$SE_{R}\left(dB\right)=20log\left(\frac{{\left(Z+Z_{0}\right)}^{2}}{4ZZ_{0}}\right)$$

(3) $$R=1-10^{\frac{-SE_{R}}{10}}$$

In the gradient structure, σ of the entry layer is deliberately minimized, resulting in the intrinsic impedance *Z* approaching that of free space (*Z*_0_). Consequently, SE_R_ and R are approaching 0, according to Equations (2) and (3), thereby suppressing reflection at the air–material interface. This improved impedance matching is essential for enabling absorption‐dominant EMI shielding behavior.

Once the EM waves enter the composite, the continuous increase in conductivity along the thickness direction facilitates progressive attenuation of electromagnetic energy. This attenuation within the composite arises from the dielectric loss mechanisms, including Ohmic and polarization losses [53, 54]. In the Ohmic loss mechanism, the interconnected MXene nanosheets form continuous conductive networks that facilitate charge‐carrier transport under an alternating electromagnetic field, leading to conduction loss via electron migration, hopping, and the generation of induced currents [55]. These currents dissipate as Joule heat due to intrinsic resistance, thereby contributing significantly to energy attenuation. In the mechanism of polarization loss, both dipolar and interfacial polarization contribute [56]. The abundant surface functional groups on MXene nanosheets introduce dipoles that attempt to reorient in response to high‐frequency electromagnetic fields, leading to polarization relaxation‐induced energy dissipation [57]. In addition, the heterogeneous interfaces among MXene nanosheets, polymer skeletons, and air‐filled pores initiate the interfacial polarization. In this mechanism, localized charges accumulate at the interfaces between different materials under alternating fields, thereby facilitating energy dissipation [58].

The hierarchical porous structure also plays a crucial role by inducing multiple reflections and scattering of electromagnetic waves within the internal cavities, thereby prolonging the propagation path and increasing the probability of interaction between electromagnetic waves and lossy media [59]. Furthermore, the spatial conductivity gradient enables progressive attenuation, where electromagnetic waves undergo continuous energy dissipation as they propagate from low‐loss to high‐loss regions. This cooperative effect ensures that most incidents of electromagnetic energy is effectively converted to thermal energy, resulting in superior absorption‐dominated EMI shielding performance.

Notably, when EM waves are incident from the high‐conductivity side, the severe impedance mismatch leads to dominant reflection, preventing effective wave penetration. This directional dependence highlights the unique advantage of the continuous gradient design in achieving asymmetric and absorption‐dominant EMI shielding.

### 3.6 Structural Stability of the Composite Under Cycling Compression

The structural stability of the composite under mechanical deformation is crucial for its practical application. To evaluate this, the as‐prepared gradient composite fabricated under the specific conditions (T = 50°C, RH = 50%, V = 0 m s^−^^1^, and C = 7 mg mL^−^^1^) was selected as a representative sample for cyclic compression testing. As shown in Figure S11a, the stress‐strain curves over repeated compression‐loading and unloading cycles (1, 50, 100, and 500 cycles) at a fixed maximum strain of 40% exhibit highly consistent profiles, with only a slight reduction in stress at a given strain after prolonged cycling. Notably, the overall curve shape remains relatively stable after 500 cycles, indicating that the gradient architecture effectively preserves its elastic deformation capability and resists structural collapse. The absence of significant hysteresis evolution or permanent deformation further confirms the excellent fatigue resistance and recoverability of the composite.

Importantly, the EMI shielding performance, after repeated compression (Figure S11b,c) at the maximum strain of 40%, shows negligible variation in shielding effectiveness (SE_A_ and SE_R_) and power coefficients (A and R) with increasing cycle numbers. This stable electromagnetic response indicates that the conductive pathways and gradient architecture remain well preserved during cyclic deformation. The strong correlation between mechanical resilience and EMI stability highlights the reliability of the composite for practical applications, where materials are often subjected to repeated mechanical stress while required to maintain consistent shielding performance.

## 4 Conclusion

In summary, we have developed a facile unidirectional evaporation strategy to fabricate MXene‐decorated melamine foams with a continuous, tunable electrical conductivity gradient for absorption‐dominant EMI shielding. Unlike conventional layer‐by‐layer approaches that produce stepwise conductivity profiles by stacking discrete layers, this method generates a monolithic gradient architecture without obvious interlayer boundaries. As a result, the composite achieves smoother impedance matching, more continuous electromagnetic attenuation, and improved structural continuity compared with conventional stepwise designs. The gradient formation is governed by the synergistic interplay of capillary, gravity, and Marangoni forces during directional solvent evaporation, which regulate the redistribution of MXene nanosheets within the three‐dimensional foam skeleton.

By tuning temperature, humidity, and MXene concentration, the conductivity gradient can be tailored to optimize impedance matching and electromagnetic wave dissipation. Under optimal conditions, the resulting composite delivers a stable SE_T_ of 53.84 dB across the X‐band with an ultralow SE_R_ of 0.032 dB and a high absorptivity of 0.99276, even after 500 compression cycles. These results demonstrate that the present method not only simplifies fabrication relative to multistep layer‐by‐layer assembly but also provides a practical route to fabricate continuously gradient porous composites for advanced low‐reflection EMI shielding applications.

## Funding

This research was funded by Advanced Research and Innovation Center (ARIC) under Project ID: 8436010, and the Research & Innovation Center for Graphene and 2D Materials (RIC‐2D) under Project ID: DP4‐8434000508, Khalifa University of Science and Technology.

## Conflicts of Interest

The authors declare no conflicts of interest.

## Supporting information




**Supporting File**: advs75732‐sup‐0001‐SuppMat.docx.

## Data Availability

The data that support the findings of this study are available from the corresponding author upon reasonable request.
