# Supplementary material for: Unidirectional Evaporation‐Induced Tunable and Continuous Gradient Composite Structure for Absorption‐Dominant Electromagnetic Interference Shielding
Source: Adv Sci (Weinh). 2026 May 15;13(45):e75732. doi: 10.1002/advs.75732 (PMC13335816; doi:10.1002/advs.75732)
Supplement: Supplementary file 1 [file ADVS-13-e75732-s001.docx]

# Supplementary Information

**Unidirectional evaporation-induced tunable and continuous gradient composite structure for absorption-dominant electromagnetic interference shielding**

Figure S 1. Characterization of the synthesized Ti₃C₂Tₓ MXene (a) XRD patterns of the MAX phase and Ti₃C₂Tₓ MXene. (b) Raman spectrum of Ti₃C₂Tₓ MXene. (c) XPS survey spectrum of Ti₃C₂Tₓ MXene.

Ti₃C₂Tₓ MXene was prepared by selectively etching the Al layers from the Ti₃AlC₂ MAX phase, producing a layered structure with rich surface terminations. The phase transformation was confirmed by X-ray diffraction (XRD) (Figure S 1a). Compared with the precursor, the (002) peak shifted from 9.7° to ~6.8° after etching, reflecting an expanded interlayer spacing resulting from Al removal and surface functionalization. The disappearance of the (104) peak further indicates the complete elimination of Al and the successful formation of Ti₃C₂Tₓ. Raman spectra (Figure S 1b) show characteristic bands in the 100–800 cm⁻¹ region, corresponding to Ti–C vibrations and termination-related modes. No signals associated with titanium oxides are detected, suggesting that the MXene retained its carbide structure without significant oxidation during the synthesis process. X-ray photoelectron spectroscopy (XPS) analysis (Figure S 1c) was employed to examine the elemental composition and surface chemistry. The survey spectrum reveals Ti and C as the main components, together with –O and –F surface terminations, while the absence of Al peaks confirms high phase purity.

Figure S 2. Schematic illustration of the sample support used in the unidirectional evaporation setup. The composite sample is elevated above the base plate by vertical supports, allowing only the bottom surface to be exposed to ambient air while minimizing lateral airflow interference, thereby enabling controlled unidirectional evaporation.

Figure S 3. Photographs comparing the pristine melamine foam and the MXene-modified composite after evaporation drying, demonstrating volume shrinkage. MF-1 corresponds to the gradient structure, while MF-2 represents the control sample for comparison. (a) Original dimensions of MF-1 and MF-2. (b) Lateral dimensions of MF-1 and MF-2 after drying. (c) Heights of MF-1 and MF-2 after drying.

Macroscopic volume shrinkage occurs during drying. The surface tension of the liquid film exerts a contracting force on the MF skeleton, drawing adjacent struts closer together as water evaporation proceeds. As shown in Figure S 3, the pristine MF reference sample (MF-2) has an initial thickness of 10 mm and lateral dimensions of 30 mm × 30 mm, while MF-1 exhibits the same dimensions prior to processing (Figure S 3a). After dip-soaking and complete drying with all surfaces exposed to air, the composite sample shrinks to a thickness of 8.5 mm and lateral dimensions of 27 mm × 27 mm, corresponding to an overall volume reduction of approximately 31% (Figure S 3b and c).

Figure S 4. (a) Optical image of the MXene/MF composite prepared at an evaporation temperature of 100°C. (b) Corresponding SEM image highlighting the severe agglomeration of MXene nanosheets (indicated by the yellow dashed circles) induced by excessive evaporation at high temperature.

Figure S 5.(a) Optical image of the MXene/MF composite fabricated using an MXene dispersion concentration of 1 mg mL⁻¹. (b) Corresponding EDS elemental mapping of Ti, illustrating the laminated distribution at low concentration.

Figure S 6. (a) Optical image of the MXene/MF composite fabricated using an MXene dispersion concentration of 11 mg mL⁻¹. (b) Corresponding EDS elemental mapping of Ti, illustrating the gradient distribution at high concentration.

Figure S 7. Effect of relative humidity (RH) on the electrical conductivity gradient profiles of the MXene/MF composite foams. (a) Conductivity profile obtained at a low RH of 5%, showing an unstable gradient with large fluctuations. (b) Conductivity profile obtained at a high RH of 100%, exhibiting a suppressed gradient due to inhibited solvent evaporation. The solid lines represent fitted curves, and the shaded regions indicate the 95% confidence bands.

Figure S 8. Cross-sectional optical images of the MXene/MF composite prepared at different relative humidity (RH). (a) Region exhibiting local flow instability and nonuniform MXene distribution at the RH of 5%. (b) Region showing the MXene distribution at the RH of 100% before freeze-drying.

Figure S 9. Photograph of the EMI testing setup. (a) The vector network analyzer configuration with labeled port numbers and the sample holder used for measurement. (b) The inner dimension of the sample holder.

Figure S 10. Photograph of a representative composite fabricated using the conventional layer-by-layer assembly method, in which MF slices with different MXene loadings were sequentially stacked to form a stepwise gradient structure.

Figure S 11. Mechanical properties of the composite with a gradient structure. (a) Cyclic compression stress-strain curves of the composite were measured over multiple loading and unloading cycles (1, 50, 100, and 500 cycles) at a fixed maximum 40% strain. (b) The EMI shielding performance (SEA and SER) after different numbers of compression cycles at a fixed maximum strain of 40%. (c) The EMI shielding performance (A and R) after different numbers of compression cycles at a fixed maximum strain of 40%.

**Supplementary Note 1. Experimental control on drying time, air flow, PI sealing, and conductivity measurement.**

**Drying time:** The drying process was continued until complete solvent removal, as confirmed by reaching a stable sample weight. The drying time depends on the processing conditions, including temperature, relative humidity (RH), and airflow (as studied and presented in Figure 4). Under standard conditions (25 °C, 50% RH, and no external airflow), the drying process typically requires approximately 13 h. In addition, higher temperatures, higher wind speeds, or lower relative humidity will need a shorter drying duration.

**Airflow control:** Airflow during the drying process was regulated using an air blower (FORMULE 5800 model) by changing its speed controller and distance from the sample. The airflow velocity was monitored with an airflow meter placed close to the sample to reflect the local conditions during evaporation.

**PI sealing method:** Polyimide (PI) tape (3M, KA03D34, thickness: 0.03 mm) was cut and wrapped around the sample to seal all surfaces except the bottom face. Multiple layers were applied to ensure effective sealing. The thin PI tape provides good conformal contact with the sample surface, thereby preventing unintended solvent evaporation from the sealed faces. In cases where minor portions of the sealed area remained exposed, a minimal amount of epoxy glue was applied.

**Conductivity measurement.** The electrical conductivity of the sliced samples (thickness = 1 mm) was measured using the four-point probe method in the in-plane direction. We note that, due to the porous nature of the samples, the calculated conductivity represents an effective electrical conductivity [1], which is suitable for comparing the relative variation along the thickness direction rather than determining absolute values. The sample geometry was set to “Rectangular” during the measurement, with the long and short sides defined by the lateral dimensions of the test specimen. The thickness was determined from cross-sectional SEM images of the specimen, typically from the representative cross-section shown in Figure 3d-iii. To ensure consistency, all measurements were performed under the consistent probe contact conditions, with the probes inserted approximately 0.2 mm into the sample from the top surface to maintain stable electrical contact while minimizing structural deformation. For each slice, measurements were taken five times, and the average value was reported.

Table S 1. Summary of EMI shielding performance of the gradient structure developed in this work and others reported in the literature.

| **Material** | **Structure type** | **Frequency band** | **Thickness (mm)** | **Density (g/cm^3^)** | **EMI SE_T_ (dB)** | **Absorptivity** | **SSE**  **(dB·cm^3^/g)** | **Ref.** |
| --- | --- | --- | --- | --- | --- | --- | --- | --- |
| MXene/CFf/TPU | Film | 8.2-12.4 | 0.5 | 1.25 | 40.4 | 0.9 | 32 | [2] |
| MXene/CNT/PVA/CA | Film | 8.2-12.4 | 0.015 | 0.14 | 51.6 | 0.98 | 371 | [3] |
| MXene/BC | Film | 8.2-12.4 | 0.004 | 0.32 | 37.3 | 0.68 | 116 | [4] |
| Annealed MXene/epoxy | Film | 8.2-12.4 | 2 | - | 41 | 0.72 | - | [5] |
| Bridged MXene | Film | 0.3-18 | 0.003 | 0.3 | 56.4 | 0.99 | 187 | [6] |
| Pure MXene | Film | 8.2-40 | 0.0023 | 0.013 | 45 | 0.78 | 3553 | [7] |
| MXene/ANFs | Aerogel | 8.2-12.4 | 1.9 | 0.08 | 56.8 | 0.75 | 693 | [8] |
| MXene/cellulose | Aerogel | 8.2-12.4 | 2 | 0.06 | 72.9 | 0.24 | 1200 | [9] |
| CNF/FeCo-LDO/rGO composite | Aerogel | 8.2-12.4 | 5.8 | 0.0031 | 65 | 0.32 | 2096 | [10] |
| CNF/PANI composite | Aerogel | 8.2-12.4 | 5 | 0.019 | 32 | 0.05 | 1662 | [11] |
| CNF/APP/Ti_3_C_2_T_x_ composite | Aerogel | 8.2-12.4 | 8 | 0.012 | 55 | 0.6 | 4583 | [12] |
| 3D printed MXene/ CNT/PI composite | Aerogel | 8.2-12.4 | 5 | 0.152 | 68.2 | 0.23 | 449 | [13] |
| CNT+Fe_3_O_4_-coated composite | Gyroid TPMS | 8.2-12.4 | 10 | 0.43 | 35.9 | 0.13 | 83 | [12] |
| MXene-coated composite | Gyroid TPMS | 8.2-12.4 | 10 | - | 59 | 0.15 | - | [14] |
| Graphene-integrated composite | Neovius TPMS | 8.2-12.4 | 15 | 1.8 | 75 | - | 94 | [15] |
| MXene-coated composite | Gyroid TPMS | 8.2-12.4 | 10 | 0.3 | 50 | 0.25 | 167 | [16] |
| PVDF/Fe_3_O_4_/CNT composite | Bulk | 8.2-12.4 | 4.8 | - | 44.5 | 0.15 | - | [17] |
| MXene/PVA composite | Foam | 8.2-12.4 | 10 | 0.01 | 28 | 0.41 | 2800 | [18] |
| PDMS-MXene@PANI/mPP | Foam | 8.2-12.4 | 12 | 0.05 | 39.8 | 0.31 | 796 | [19] |
| MF-MXene@AgNW-PEG | Foam | 8.2-12.4 | 5 | - | 30.5 | 0.3 | - | [20] |
| MXene/Ag@ZnO/WPU/Melamine composite | Foam | 8.2-12.4 | 4 | 0.023 | 39.9 | 0.14 | 1735 | [21] |
| Continuous gradient composite | **Foam** | **8.2-12.4** | **~10** | **0.032** | **~54** | **~0.007** | **1688** | **This work** |

## References:

[1]	F. Deng, Q. Zheng, An analytical model of effective electrical conductivity of carbon nanotube composites, Appl. Phys. Lett. 071902 (2008). https://doi.org/10.1063/1.2857468.

[2]	N. Duan, Z. Shi, Z. Wang, B. Zou, C. Zhang, J. Wang, J. Xi, X. Zhang, X. Zhang, G. Wang, Mechanically robust Ti3C2Tx MXene/Carbon fiber fabric/Thermoplastic polyurethane composite for efficient electromagnetic interference shielding applications, Mater. Des. 214 (2022) 110382. https://doi.org/https://doi.org/10.1016/j.matdes.2022.110382.

[3]	T. Zhou, C. Zhao, Y. Liu, J. Huang, H. Zhou, Z. Nie, M. Fan, T. Zhao, Q. Cheng, M. Liu, Large-Area Ultrastrong and Stiff Layered MXene Nanocomposites by Shear-Flow-Induced Alignment of Nanosheets, ACS Nano 16 (2022) 12013–12023. https://doi.org/10.1021/acsnano.2c02062.

[4]	Y. Wan, P. Xiong, J. Liu, F. Feng, X. Xun, F.M. Gama, Q. Zhang, F. Yao, Z. Yang, H. Luo, Y. Xu, Ultrathin, Strong, and Highly Flexible Ti3C2Tx MXene/Bacterial Cellulose Composite Films for High-Performance Electromagnetic Interference Shielding, ACS Nano 15 (2021) 8439–8449. https://doi.org/10.1021/acsnano.0c10666.

[5]	L. Wang, L. Chen, P. Song, C. Liang, Y. Lu, H. Qiu, Y. Zhang, J. Kong, J. Gu, Fabrication on the annealed Ti3C2Tx MXene/Epoxy nanocomposites for electromagnetic interference shielding application, Compos. Part B Eng. 171 (2019) 111–118. https://doi.org/https://doi.org/10.1016/j.compositesb.2019.04.050.

[6]	S. Wan, X. Li, Y. Chen, N. Liu, Y. Du, S. Dou, L. Jiang, Q. Cheng, High-strength scalable MXene films through bridging-induced densification, Science (80-. ). 374 (2021) 96–99. https://doi.org/10.1126/science.abg2026.

[7]	W. Huang, X. Liu, Y. Wang, J. Feng, J. Huang, Z. Dai, S. Yang, S. Pei, J. Zhong, X. Gui, Ultra‑Broadband and Ultra-High Electromagnetic Interference Shielding Performance of Aligned and Compact MXene Films, Nano-Micro Lett. 17 (2025) 1–13. https://doi.org/10.1007/s40820-025-01750-z.

[8]	Z. Lu, F. Jia, L. Zhuo, D. Ning, K. Gao, F. Xie, Micro-porous MXene/Aramid nanofibers hybrid aerogel with reversible compression and efficient EMI shielding performance, Compos. Part B Eng. 217 (2021) 108853.

[9]	Z. Zong, P. Ren, Z. Guo, J. Wang, Z. Chen, Y. Jin, F. Ren, Three-dimensional macroporous hybrid carbon aerogel with heterogeneous structure derived from MXene/cellulose aerogel for absorption-dominant electromagnetic interference shielding and excellent thermal insulation performance, J. Colloid Interface Sci. 619 (2022) 96–105.

[10]	M. Ma, Y. Liao, H. Lin, W. Shao, W. Tao, S. Chen, Y. Shi, H. He, Y. Zhu, X. Wang, Double-layer of CNF / rGO film and CNF / rGO / FeCo-LDO aerogel structured composites for efficient electromagnetic interference shielding, Carbon N. Y. 220 (2024) 118863. https://doi.org/10.1016/j.carbon.2024.118863.

[11]	A.R. Pai, T. Binumol, D.A. Gopakumar, D. Pasquini, B. Seantier, N. Kalarikkal, S. Thomas, Ultra-fast heat dissipating aerogels derived from polyaniline anchored cellulose nano fi bers as sustainable microwave absorbers, Carbohydr. Polym. 246 (2020) 116663. https://doi.org/10.1016/j.carbpol.2020.116663.

[12]	Y. Zhang, J. Yu, J. Lu, C. Zhu, D. Qi, Facile construction of 2D MXene (Ti3C2Tx) based aerogels with effective fire-resistance and electromagnetic interference shielding performance, J. Alloys Compd. 870 (2021) 159442. https://doi.org/10.1016/j.jallcom.2021.159442.

[13]	T. Xue, Y. Yang, D. Yu, Q. Wali, Z. Wang, X. Cao, 3D Printed Integrated Gradient ‑ Conductive MXene / CNT / Polyimide Aerogel Frames for Electromagnetic Interference Shielding with Ultra ‑ Low Reflection, Nano-Micro Lett. 15 (2023) 1–14. https://doi.org/10.1007/s40820-023-01017-5.

[14]	A. Kamal, K. Liao, M.M. Syed, A. Otabil, Absorption-Dominant Electromagnetic Interference Shielding of Ti 3 C 2 T x MXene-Coated and Fe 3 O 4 -Integrated TPMS Composites with Coupled Gradient Conductivity and Size-Graded Structure, Adv. Sci. (2026) 1–14. https://doi.org/10.1002/advs.202522856.

[15]	P. Srinivas, L. Jacob, C.M. Shebeeb, H. Butt, I. Barsoum, R.K.A. Al-rub, W. Zaki, Effect of printing parameters and triply periodic minimal surfaces on electromagnetic shielding efficiency of polyvinylidene fluoride graphene nanocomposites, Addit. Manuf. 95 (2024) 104544. https://doi.org/10.1016/j.addma.2024.104544.

[16]	A. Kamal, B. Li, S.K. Siddique, D. Zhang, K.B. Shingare, A. Schiffer, Tailoring triply periodic minimal surface architectures with Ti₃C₂T ₓ MXene for high-performance absorptive EMI shielding, Adv. Compos. Hybrid Mater. 9 (2026) 1–17.

[17]	Q.M. Zhang, J. Cui, S. Zhao, A.L. Gao, G.F. Zhang, Y.H. Yan, Regulation binary electromagnetic filler networks in segregated poly(vinylidenefluoride) composite for absorption-dominated electromagnetic interference shielding, J. Appl. Polym. Sci. 140 (2023). https://doi.org/10.1002/app.53650.

[18]	H. Xu, X. Yin, X. Li, M. Li, S. Liang, L. Zhang, L. Cheng, Functional Nanostructured Materials ( including low-D carbon ) Lightweight Ti2CTX MXene / Poly ( vinyl alcohol ) Composite Foams for Electromagnetic Wave Shielding with Absorption Dominated Feature, (2019). https://doi.org/10.1021/acsami.8b21671.

[19]	X. Jia, B. Shen, L. Zhang, W. Zheng, Construction of compressible Polymer/MXene composite foams for high-performance absorption-dominated electromagnetic shielding with ultra-low reflectivity, Carbon N. Y. 173 (2021) 932–940.

[20]	Y. He, Y. Shao, Y. Xiao, J. Yang, X. Qi, Y. Wang, Multifunctional phase change composites based on elastic MXene/silver nanowire sponges for excellent thermal/solar/electric energy storage, shape memory, and adjustable electromagnetic interference shielding functions, ACS Appl. Mater. Interfaces 14 (2022) 6057–6070.

[21]	T. Zuo, W. Wang, D. Yu, MXene/Ag@ZnO/WPU/Melamine gradient composite foams prepared by a unidirectional evaporation approach for absorption-dominated electromagnetic interference shielding, J. Alloys Compd. 966 (2023) 171644. https://doi.org/10.1016/j.jallcom.2023.171644.
